# Curious Case of Clot Urinary Retention

**DOI:** 10.51894/001c.144626

**Published:** 2025-09-30

**Authors:** Upahvan Rai

**Affiliations:** 1 Department of Urology U of M Health Sparrow

## 112

### INTRODUCTION

Hematuria is one of the most common urologic pathologies. Hematuria presenting as clot urinary retention, though severe, is algorithmic in its approach and more than half of cases are managed conservatively. Here we present a case of clot urinary retention that required continuous bladder irrigation but ultimately called for an interventional radiology procedure.

### CASE DESCRIPTION

This patient originally underwent a right renal biopsy during the work up of her unexplained kidney dysfunction in the setting of numerous rheumatologic diseases. Two weeks following her biopsy she presented to an outside hospital with clot urinary retention necessitating transfer to our institution. CT imaging demonstrated an area of contrast enhancement at the right kidney concerning for extravasation. Ultimately she was found to have a pseudoaneurysm which was likely a direct result of her renal biopsy and injury to a renal vessel. She was taken urgently to interventional radiology where an angiogram was performed demonstrating large pseudoaneurysm. Given the instability and propensity for hemorrhage of these pseudoaneurysms, she successfully underwent coil embolization.

### CONCLUSIONS

At our institution, embolization procedures have been increasingly important in the management of refractory gross hematuria secondary to renal etiology. This case highlights the importance of imaging and a multidisciplinary approach of severe hematuria particularly in the ever evolving field of minimally invasive endourology.

